# Food Composition Database Format and Structure: A User Focused Approach

**DOI:** 10.1371/journal.pone.0142137

**Published:** 2015-11-10

**Authors:** Annabel K. Clancy, Kaitlyn Woods, Anne McMahon, Yasmine Probst

**Affiliations:** School of Medicine, University of Wollongong, Wollongong, NSW, 2500, Australia; Colorado State University, UNITED STATES

## Abstract

This study aimed to investigate the needs of Australian food composition database user’s regarding database format and relate this to the format of databases available globally. Three semi structured synchronous online focus groups (M = 3, F = 11) and n = 6 female key informant interviews were recorded. Beliefs surrounding the use, training, understanding, benefits and limitations of food composition data and databases were explored. Verbatim transcriptions underwent preliminary coding followed by thematic analysis with NVivo qualitative analysis software to extract the final themes. Schematic analysis was applied to the final themes related to database format. Desktop analysis also examined the format of six key globally available databases. 24 dominant themes were established, of which five related to format; database use, food classification, framework, accessibility and availability, and data derivation. Desktop analysis revealed that food classification systems varied considerably between databases. Microsoft Excel was a common file format used in all databases, and available software varied between countries. User’s also recognised that food composition databases format should ideally be designed specifically for the intended use, have a user-friendly food classification system, incorporate accurate data with clear explanation of data derivation and feature user input. However, such databases are limited by data availability and resources. Further exploration of data sharing options should be considered. Furthermore, user’s understanding of food composition data and databases limitations is inherent to the correct application of non-specific databases. Therefore, further exploration of user FCDB training should also be considered.

## Introduction

Food composition data and databases are required by a variety of different users, across multiple fields, such as dietetics, food technology, biomedical research and public health nutrition [1,2]. Common uses of food composition data include: dietary assessment, research into diet-disease relationships, food regulatory policy formation and food labelling [3]. The area of practice largely effects the requirements, level of precision and application of food composition data. In addition, the rapidly changing and globalisation of the food supply adds to the complexities of database development and use [4]. It is therefore essential, that food and nutrition professionals, in each of these fields, have an understanding of how data is generated, the differences and intended purpose of the variety of available databases and related software, that draws on the databases [5].

The Oxford Dictionary defines format (computing) as ‘a defined structure for processing, storage or display of data’[6]. Stumbo [7] suggests that food composition database (FCDB) format should match the needs of the user, and that users should understand a databases design when choosing a database for use. The European Food Information Resource (EuroFIR) network also recognises the importance of user input into FCDB development.^8^ Recently, EuroFIR have used the ‘Use Case’ approach, which aids in identification of the functional requirements of users, according to the activity the user performs [8]. The information gathered can then be used to assist in the improvement of a databases features and format [8].

EuroFIR has also recently been working to create standards for database compilation, including standard exchange formatting within European nations to allow comparison and harmonisation between individual countries databases [2,9]. A recent study by Ireland and Møller [9] examined Australian, New Zealand, US and Canadian FCDB to establish their ability to be standardised against the draft criterion for database format, developed by EuroFIR. The study identified that these databases differed in the methodology used to define and describe foods as well as how nutrient values were documented. This study was conducted from a FCDB compilation perspective and hence, consideration of the needs of FCDB users was not among the criterion addressed [9].

Additionally, the importance of user input can be seen in a study by McCabe-Sellers and Chenard [10] which investigated the requirements of a FCDB to meet the needs of US dietitians. McCabe-Sellers and Chenard [10] emphasises the importance of accessibility and availability of data, and suggests that this has been improved by technological advancements. Secondly, improved technology provides dietitians with the opportunity to convert food composition data into formats suitable for dietary planning, assessment and for nutrition information panel development [3].

Currently, in Australia, there are two main FCDB, the reference database, NUTTAB 2010 and the most recent survey database, AUSNUT 2011–13 [11, 12]. These databases are produced and maintained by Food Standards Australia and New Zealand (FSANZ). NUTTAB contains primarily analysed data on the macronutrient and micronutrient composition of Australian foodstuffs, and is therefore incomplete. AUSNUT provides a complete nutrient dataset specific to the Australian Health Survey. Unfortunately, phytochemical data is limited in both databases [11]. Currently research into whether the content and usability of these databases, meet the needs of Australian FCDB users is limited.

Quantitative data surrounding the needs of FCDB users [1, 10], as well as the challenges and limitations surrounding food composition data and databases is available at an international level [7,9]. However, the applicability of international research to the Australian context and FCDB remains unknown. Alternatively, qualitative research allows an in depth analysis of a research topic, through insight into participants thoughts, opinions and beliefs [13]. However, this methodology has not been regularly employed in relation to FCDB user understanding and needs assessment.

Therefore, this study aimed to qualitatively examine the needs of Australian FCDB users regarding FCDB format and relate this to the format of databases available globally. In a broader context, the purpose of this study is to assist in providing insight into user requirements for further database development, including the potential development of an Australian phytochemical FCDB.

## Materials and Methods

This study was part of a larger study aimed at investigating the beliefs of FCDB users, surrounding the development of a phytochemical FCDB. This study (Probst, Trip Fellowship 2014–2016, #APP1072484) was approved by the University of Wollongong Human Research ethics committee. Prior to commencing the focus groups and interviews, all participants provided written, informed consent and completed a short, non-identifiable demographic questionnaire, to gather information relating to age, gender, education and employment. Online synchronous focus groups and semi-structured telephone interview methodology were chosen to allow nationwide participation [14].

### Online focus groups

#### Recruitment

Purposive sampling was conducted by placing advertisements with nationally recognised professional organisations related to food and nutrition. Members of the organisations who responded to the advertisement were assessed for eligibility and availability. Inclusion criteria were individuals aged 18 years or above, with an interest in or users of FCDB and the ability to understand, communicate in and read English.

#### Data Collection

The beliefs of respondents, surrounding food composition were investigated through online synchronous focus group methodology. Individual test sessions for focus group participants were held prior to the focus group, to assist in reducing technical problems. Groups (3–6 participants) ran for 60–90 minutes and were conducted online using Adobe Connect 9.2 (Adobe Systems Incorporated. 2014.) with a consistent, experienced female moderator with a doctoral degree in nutrition science (AM), technical support (YP) and observer (KW), for all groups, as suggested by Krueger and Casey [15]. AM and KW were visible to the participants, whilst YP was not visible to lessen impact on group bandwidth. All focus groups were audio and video recorded. The moderator followed a semi-structured format throughout the focus groups, with questions designed according to Green and Thorogood [16]. The questions were pilot tested within the research group prior to use in the focus groups. The questions covered attitudes and beliefs surrounding food composition, such as the use, training and limitations of databases, as well as the participants understanding of phytochemicals (S1 Table). The moderator focused on clarifying points, probing and exploring themes as well as encouraging participation of less vocal members. The observer’s written record included verbal and non-verbal information to aid in distinguishing individual responses and enhancing the final analysis.

### Key informant interviews

#### Recruitment

Key informants in FCDB use and development as well as phytochemical research were identified from a review [17] of the Australian food composition program or mentioned by participants during the focus groups and interviews. Emails explaining the project were sent to participants identified as key informants inviting them to participate in an interview.

#### Data Collection

The opinions of key informants surrounding food composition were collected via 30 minute telephone interviews. The interviews were conducted by the same moderator, AM, following a semi-structured design, with questions supported by the principles of Green and Thorogood [16]. Questions covered in the interviews focused on the experiences of participants in food composition, related to their area of expertise (S2 Table) and were of a similar nature to those used in the online focus groups.

#### Data analysis

Focus groups and interviews were recorded digitally and spoken words were transcribed verbatim. Analysis of video recordings was outside the scope of this project. Transcripts were reviewed against recordings by a researcher (AC or KW) not involved in the data collection, to ensure accuracy. Grounded theory was used to guide data coding and analysis [18]. The primary coder (AC) carried out initial content and thematic analysis to identify dominant themes. Sub-categories were identified within themes to differentiate responses. A secondary coder (KW) similarly coded the data and variances were identified and deliberated to reach consensus. All themes and variations were reviewed with QSR NVivo 10.0 qualitative software (QSR International Ltd. Doncaster, Vic., Australia). Further thematic analysis was performed by AC and KW, using an iterative approach. Exemplar quotes illustrating each theme were also identified. A flowchart of the analysis process can be viewed in S1 Fig.

For the purpose of the present study a focus was given to database format and usefulness in practice. Schematic analysis was applied to dominant themes to identify major themes related to database format specifically.

### Desktop analysis

Desktop analysis involved examination of the format of six FCDB. Aside from the major Australian databases, NUTTAB 2010 and AUSNUT 2011–13 [13], databases from the US [19], UK [20], Canada [21] and NZ [22] were included as they are current, publically available, published in English and likely to be used by Australian FCDB users due to similarities in their food supply systems [11, 19]. The formal website of each country’s database was used to assist in gathering information surrounding format, related to the themes extracted from schematic analysis. The webpage and supporting documents were examined to extract details. Relevant components of each database were identified and tabulated.

## Results

Of the n = 23 participants who expressed interest in the study, three men and eleven women (61%, n = 14), of ages ranging from 24 to 69 years participated in three online focus groups (n = 6, 3, and 5 participants respectively). Eight key informants were invited to take part in an interview, and six female key informants participated in an interview (Table 1). Reasons for participant drop-out included time and day of focus groups, the length (90 minutes) of the focus group, and the inability to link with Adobe Connect. Two participants typed responses to enable them to participate in the focus groups.

**Table 1:** Descriptive data of total sample of participants (n = 20).

Characteristics	Focus Groups	Interviews
**Number of subjects**	14	6
**Gender**:		
***Male***	3	0
***Female***	11	6
**Age (years)**:		
***20–34***	9	2
*35–49*	2	1
*50+*	3	3
Highest Level of Education:		
***Bachelor***	4	1
***Masters***	6	1
***PhD***	4	4
Current Area of Practice		
***Clinical dietetics***	4	1
***Academic and/or Researcher***	3	2
*First year graduate*	1	0
***Doctoral candidates***	5	0
***Public health nutrition***	1	1
***Food and/or health agency***	0	2
Geographical distribution		
***New South Wales***	12	5
***Victoria***	2	0
***Canberra***	1	1

From thematic analysis of 149 pages of transcribed data, 24 dominant themes were identified (S1 File). From schematic analysis (S2 Fig) five major themes (italicised in the text) were found to be related to database format (Table 2) as they best represented and aligned with the previously defined definition of format [6]. These five major themes characterised the overall structure of FCDB.

**Table 2:** Summary of five major format related themes identified from schematic analysis of 24 dominant themes, extracted from focus groups and interviews.

Major themes (definition)	Sub-categories (definition)	Exemplary quote
Database use (Current use of FCDB)	-type (which FCDB are used)	“Two of our reference databases NUTTAB 2006 and NUTTAB 2010, as well as two of our service specific databases AUSNUT 2007 and the more recent one related to the Australian Health Survey…AUSNUT 2011–13.” (Interview 2)
	-purpose (what are FCDB and data used for)	“I mean to a large extent the nutrient databases are linked to what we know about nutrient requirements …. the components that we think are important for health ……….then you need to have the composition database to be able to assess diets against those things” (Focus group 2)
	-usability (ease of use, intuitiveness and learnability for tertiary educated individuals including how they interpret descriptors and use the data)	“So say you’ve finished doing a research project, and then you’re only using it …. a couple of times a year ……..you do get worse at it and forget the detail, and yeah it’s a little bit more difficult…….. but what it does mean to me is that it’s not intuitive” (Focus group 2)
	-database choice (reasons for choosing a FCDB)	“I’m pretty inexperienced in deciding which one to use… I think I normally use AUSNUT …for most of my purposes …. but to be honest … I don’t really know the pros or cons of either one” (Focus group 3)
Framework (Presentation of data including design, layout, structure, food labelling as well as technological components including software, applications and functions of the data)		"I know a standard format at the moment is Excel……as a researcher I’m much more comfortable with that, than I am with any other format because otherwise you have to modify it to… your system….. I’ve never had a problem, I’ve never really thought of it as a problem even if it’s in a format that you don’t really like. Anyone with a, well, I, anyone who’s working in databases probably can manipulate it however they want” (Interview 5)
Food classification (How foods are grouped and named in relation to database format and use)		“I’ve watched a lot of, a lot of students use the …. FoodWorks software… which uses AUSNUT and watching the challenges …. on being able to just follow names or find those foods …. because it’s just so difficult to navigate …. I think that’s….. going to be a challenge with any database, let alone an Australian databases, uh in naming in an easy way” (Focus group 1)
Accessibility and Availability (Ability to retrieve data related to location, ownership and resources)	-central repository (a centralised system for all food composition data to be located and collaborated)	“Pooling that data is a valuable, is a power in numbers if you like and at the moment that’s what we need to get this momentum in terms of volume of information but having said that I think um we need to ensure that around that there are certain standards” (Focus group 3)
Data derivation (Analytical techniques used to obtain food composition data, including standardised techniques)		“Conscious of trying to ensure the quality of the numbers, and so they’re not always very ready to accept data that, particularly published data unless they’re really confident in the sampling and the analytical methodologies that are used” (Focus group 2)

The thematic category of *database use*, the processes involved in using and choosing the database was identified as a key format related theme, with a close link to the theme of awareness of limitations. The theme of database *framework* included the display of data, as well as the functions of the data, especially in relation to dietary software. Thirdly, the theme of *food classification*; the description of food and nutrients was identified as a key component of database format. Additionally, the theme of *accessibility and availability* of data, related to storage, retrievability and ownership of data and databases, was recognised as associated with format. This included the idea of a central repository; a centralised system for all food composition data to be located and collaborated. Lastly, the theme of *data derivation* describes the variety of methods used to obtain the data for a FCDB. These methods have a strong link with the underlying themes of resources and data accuracy and reliability.

The desktop analysis of six key FCDB format (Table 3) revealed that the USDA Nutrient Database for Standard Reference was the largest (n = 8618 foods) and most recently updated database [19]. NUTTAB 2010 [11] and the NZ Food Files (unabridged) database [22] were the only two databases based on primarily analysed data. Food classification systems varied between all six databases. However, all databases had supporting documentation available to describe food descriptors and data derivation. Microsoft Excel was a common format available between all databases. The software available for manipulation of databases varied between countries.

**Table 3:** Desktop analysis and examination of six key food composition databases format.

Background information	Name of database	NUTTAB	AUSNUT	USDA National Nutrient Database for Standard Reference	NZ FOOD files (unabridged)	UK Composition of Foods Integrated dataset	Canadian Nutrient file
	**Current version**	2010	2011	2014	2013	2015	2010
	**Country of Origin**	Australia	Australia	USA	New Zealand	UK	Canada
	**Funding source**	FSANZ^a^	FSANZ^a^	USDA^b^	Plant & Food research (ltd) & Ministry of Health	FSA^c^	Health Canada
	**Number of foods**	2668	5644	8618	2580	3423	5807
	**Number of food components**	245	51	150	360	186	150
	**Phytochemical information?**	Some carotenoids	No	No^d^	Some carotenoids	Yes-phytosterols^e^	Some carotenoids
**Database use**	**Type**	Reference	Survey	Reference	Reference	Reference	Reference
	**Purpose**	Provide analysed nutrient data for Australia	Nutritional analysis of NHS^f^ data	Major source of food composition data for the US	Major source of food composition data for NZ	Major source of food composition data for the UK	Major source of food composition data for Canada
**Framework**	**Layout**	Tables	Tables	Tables	Tables	Tables	Tables
	**Software Available**	FoodWorks 7^g^	FoodWorks 7^g^	FoodWorks 7^g^ Nutribase professional Food Processer SQL Nutritionist Pro^h^ CANDAT^i^	FoodWorks 7^g^	WISP^j^, Microdiet^k^, Dietplan^l^, Crisp^k^, Nutmeg, Nutricalc, Compeat^m^	Nutribase professional^n^ CANDAT^i^
	**File Type(s)**	Txt.^o^Tab.^p^, Excel.^q^	Excel.^q^	Access^r^, ASCII^s^, Excel^q^	ASCII^s^, Excel.^q^	Excel.^q^, ASCII^m^	DBF^t^***
**Food classification**	**Number of Food groups**	20	23	25	22	23	25
	**Food nomenclature system used**	Based on COFA^v^ food identification INFOODS^w^ food description	2011–2013 AHS classification system	Langual food description	INFOODS^w^ food description	Not specified****	INFOODS^w^ food description Bilingual
	**Food descriptors explanation?**	Yes	Yes	Yes	Yes	Yes- limited^x^	Yes
**Data derivation**	**Data collection method(s)**	Primarily analysis	Compilation	Compilation	Primarily analysis	Compilation	Compilation
	**Supporting document(s)?**	Yes	Yes	Yes	Yes	Yes	Yes
**Accessibility and availability**	**Online search option?**	Yes	No	Yes	No	No	Yes
	**Where available?**	FSANZ^a^ website^y^ ^(FSANZ 2013)^	FSANZ^a^ website^z^ ^(FSANZ 2013)^	USDA^b^ website^aa^ ^(USDA 2014)^	www.foodcomposition.co.nz/ ^(NZIPFR 2014)^	FSA^c^ website ^bb^ ^(FSA 2010)^	Health Canada^cc^ ^(Health Canada 2010)^

^a^ Food Standards Australia and New Zealand,

^b^ United States Department of Agriculture,

^c^ Food Standards Agency,

^d^ Separate databases for flavonoids, carotenoids, proanthocyanidins and isoflavones,

^e^ Eurofir EBASIS contains bioactive data for UK and Europe,

^f^ National Health Survey,

^g^ https://www.xyris.com.au/foodworks/fw_pro.html,

^h^ http://www.nutribase.com/highend.html,

^i^ http://www.foodresearch.ca/wp-content/uploads/2013/06/candat-features-1.pdf,

^j^ Tinuviel Software,

^i^ Downlees Systems,

^k^ Forestfield Software,

^l^ Kelicomp,

^m^ http://www.tinuvielsoftware.com/faqs.htm,

^n^ http://www.dietsoftware.com/canada.html,

^o^ Text file: a file that only contains text,

^p^ A file containing tables of information stored in columns and separated by tabs (can be exported into almost any spreadsheet program),

^q^ Microsoft Excel spreadsheet,

^r^ Microsoft Access Database file: is a database file with automated functions and queries,

^s^ American Standard Code for Information Interchange (a standard file type that can be used by many programs),

^t^ Database File Format (this file type can be opened with Microsoft Excel and Access),

^u^ information to create Excel or PDF available,

^v^ Composition of Foods, Australia,

^w^ International Network of Food Data System,

^x^ Users guide states food name is most descriptive & recognisable of food referenced

^y^ http://www.foodstandards.gov.au/science/monitoringnutrients/nutrientables/nuttab/Pages/NUTTAB-2010-electronic-database-files.aspx,

^z^ http://www.foodstandards.gov.au/science/monitoringnutrients/ausnut/ausnutdatafiles/Pages/default.aspx,

^aa^ http://ndb.nal.usda.gov/ndb/search/list,

^bb^ http://tna.europarchive.org/20110116113217/ http://www.food.gov.uk/science/dietarysurveys/dietsurveys/,

^cc^ http://webprod3.hc-sc.gc.ca/cnf-fce/index-eng.jsp

## Discussion

Online focus groups and interviews were an effective way to explore participant’s thoughts surrounding food composition.

The needs and challenges of Australian FCDB users identified from qualitative analysis are similar to those reported internationally [1, 2, 5, 7, 8]. It is apparent that despite the improved technology and standards in the area, professionals still face many challenges in the use of food composition data and databases.

### Database use

Database use and its capacity to meet the needs of the user were reported to be influenced by the type, purpose, choice, and usability of a database. This study identified that due to the wide variety of uses and users of FCDB, finding or developing a database to meet the needs of all professionals is challenging.

“We have so many different users that have such different needs; it’s really difficult to develop… a product that really meets all of them. So there’s always … compromises and frustrations for users” (Interview 2)

This is supported by Egan et. al. [3] who states that as the users and applications of FCDB are largely varied; this presents a significant issue when identifying standards for format. Conversely, from a user’s perspective this makes database choice and usability equally challenging.

An Australian example identified in the focus groups and interviews is that the primary purpose of FSANZ is in food regulation. Hence, the development of food composition data is driven by regulatory needs. This creates constraints for users of this data for purposes other than regulation of food standards or the Australian National Health Survey [11].

Furthermore, this reiterates the importance of understanding a databases purpose, and hence it’s likely limitations [5]. Additionally, this study has highlighted that many database users are reportedly not conversant in making informed choices, as to which database to use, as well as understanding the differences between a database and its supporting software. Whilst many studies [12, 23–25]. provide supporting information on how to make an appropriate database choice, it is apparent from this study that further training is likely still required. This is further supported by McCabe-Sellers and Chenard [10] who came to a similar conclusion when examining the FCDB needs of US dietitians.

### Framework

Data presentation, database layout and software components and functions were all closely linked with formatting. Few concerns were raised about the database layout from a users’ perspective, in this study. Participants were generally comfortable with the table (columns and rows) design and the associated file formats e.g. Excel. It is also likely that user’s associate software design with FCDB and do not differentiate the two tools. However, in contrast from a FCDB development perspective:

“Making decisions about whether to combine data sets, replace data sets ….. about how to actually present the information to the public…are huge challenges” (Interview 2)

This is supported by Stumbo [25] who highlights that how a software program summarises data entry, food components and meals were an important consideration. Secondly, ensuring the output generated is presented in a way that is meaningful to users is critical [25, 26]. It must be emphasised, that users need to understand the difference between software and the FCDB, which the software draws on.

One suggestion for data presentation that arose from this study was to provide a range for each data value rather than one number. The purpose being to better represent and emphasise the variability of nutrients in food, especially phytochemicals. However, a range could present difficulties when calculating and applying data for use. Pennington et. al. [5] suggests that by providing the standard deviation and information on the number of samples analysed, users may obtain a better understanding of the data. Additionally, from the literature, the search functions of a software package were identified as a key framework component, which is also tightly linked with food classification [23, 25, 27].

### Food classification

How foods are grouped and named in a food composition database was highlighted as a major theme in this study. Interpretation of food descriptors presented a challenge to many participants, as identified by the quote below.

“actually understanding how users, use the data, is important I think in looking at the descriptions, often I think maybe there isn’t enough, consultation with the users about the descriptors and maybe that’s something that could be used to make it better and …simpler for people to use than it has been in the past” (Focus group 2)

Similarly, Charrondiere and Burlingame [28] identified the accurate and unambiguous description of food as an important component of FCDB, noting that descriptors commonly include a unique abbreviation, definition, common and scientific names of a food item [28]. Stumbo [7] also identified food description as an area of difficulty in database structure. Stumbo [7]explains that food nomenclature differs depending if a database is a reference or survey database. This was further highlighted in the desktop analysis (Table 3), where food nomenclature systems were found to differ between reference and survey databases, as well as between countries. Additionally, alternative names, language and spelling can be a limitation [2, 25, 29]. Many food nomenclature systems are currently available, such as LanguaL, [29, 30]. These systems aim to make database searching and food classification more user friendly. However, these systems have yet to be fully incorporated into an Australian database [9, 30]. Though it should be noted that FoodWorks software (Xyris Software Australia PTY. LTD.), which draws upon NUTTAB and AUSNUT databases, utilises INFOODS tagnames. Additionally, a recent reflection on the new Czech FCDB identified that visual descriptors may be an effective and simple means to make a databases written food descriptions more user friendly [31].

### Accessibility and Availability

Accessibility and availability of food composition data and databases can be influenced by location and proprietorship. The idea of a central repository, a place where all food composition data, from all sources could be stored and accessed produced mixed responses from participants. One common viewpoint was the capability of a central repository to improve the amount of readily available data and the associated advantages.

“I think it is ….. an important thing to have it compiled in the one spot and easy to access … for all people using… that information” (Interview 4)

However, in contrast, concern was raised surrounding resources, data access, standards and accuracy, including possible misinterpretation, of pooled data. Internationally, an example of a central repository is the USDA Nutrient Databank System [32]. This system methodically captures and aggregates nutrient information from various sources, such as lab analyses, food industry and scientific literature. This information is then used to provide data to many US databases, including the Standard Reference database and the My Pyramids Equivalent database [32]. Other examples include; EuroFIRs FoodEXplorer online tool allows users to simultaneously search 28 national FCDB’s [2]. Whilst, this multicentered system provides a more cost-effective strategy for international data distribution, than a single multinational system, it relies on all participating databases proprietors maintaining the quality of their individual FCDB’s [33]. Additionally, it may be beyond the needs of the average FCDB user.

Two index systems are also available internationally, the International Nutrient Databank Directory [23], managed by the US National Nutrient Databank conference and the International Food Composition Tables Directory [23], upheld by INFOODS. These two systems list available FCDB and software packages of various companies and governments, with the aim to assist users in choosing the most appropriate FCDB for their needs [23].

### Data derivation

Data derivation methodology and associated data quality was an area of importance highlighted in this study. This included consideration of sampling and analytical techniques, data imputation as well as clearly documented analyses.

“It’s like a gold standard … its very time consuming and very expensive …. all the different places you have to sample and the different food outlets … to get the data for the database” (Focus group 1)

Stumbo[7] also noted that a key component of database structure is data derivation. Similarly, a study by Egan et al. [3] found analytical methods and documentation of methods to be important components of standard database criterion.

The quality and accuracy of data was found to be largely associated with methodology in this study. Concern was raised around the types of methods chosen and the use of imputed data.

“borrowing data from other countries… particularly borrowing only some of the data… I don’t think that’s… an accurate way of doing things” (Interview 5)

It was also expressed that careful consideration of the variability of a foods composition should be applied when choosing analytical techniques and imputing data, as well as understanding any associated database limitations. This further reiterates that the analytical methods used to obtain food composition data greatly influence the intended use and application of a FCDB, especially as data quality and accuracy are inherent to the reliability of dietary interventions, nutrition policies and research into diet-disease relationships [12]. Various methods of analysis, imputed data and recipe calculations are commonly used to provide food and nutrient data amongst all the current national databases [34]. Transparent documentation of assumptions and methods applied is therefore crucial to database users, and hence, should be incorporated as part of any database development and publishing, especially in relation to food fortification [34]. Finglas et al. [2] also notes that the desire for complete datasets has led to increased borrowing of data, and hence information on the data’s origin is crucial to ensure the borrowed data is appropriate. Currently, the national databases all provide supporting documentation. It is unclear from this study if these documents are regularly sought and used, highlighting an area for further research. Reasons for not using supporting documents may be: time poor or difficulty locating, especially if using dietary software, which does not highlight these limitations. Additionally, user understanding of the data and databases and awareness of supporting documentation may also limit use. EuroFIR’s FoodEXplorer online tool returns information on both the food item and its supporting documentation, for example; method of analysis. This is an ideal method to ensure all users are aware of supporting documentation and data derivation [2].

Another common viewpoint expressed in relation to methodology was the cost and time required to produce accurate data, as well as the resources required to use and develop the gold standard techniques.

“It also depends on … resources, time …because some of the…ideal methodologies are just not accessible for small group research” (Focus group 1)

Resources, particularly expenses are identified as a limitation by Harrison [34]. Funding is essential to maintaining, updating, developing and expanding FCDB in line with evolving knowledge in nutritional epidemiology, and diet disease relationships [34].

Guest et. al. [35] suggests at least six interviews are required for theoretical saturation of results. This was achieved in this study with six key informant interviews, complemented by three focus groups. However, despite this, we acknowledge that participation numbers were still small, and participants were mostly from New South Wales and were dietitians. Therefore, caution should be taken when extrapolating these results to users other than Australian dietitians. The low number of respondents may have been due to placement of advertisements in newsletters, which was largely outside the researcher’s control. [35] A longer recruitment period or conducting the focus groups in the evening or on the weekend may be useful for increasing participation rates in the future. Consideration should also be given to patient gifts for participation.

The use of online focus groups was beneficial in its potential to allow nation-wide participation and to include a variety of practices areas. However, online focus group methodology also presented difficulties in observing non-verbal cues, e.g. hand gestures and participant-participant interactions, key in face-to-face focus groups [36]. Technological issues such as time delays, compatibility of software programs and internet broadband also limited participant interactions and in some cases involvement in the study. Technical support (YP) typed suggestions to participants to assist in improving audio visual quality. Transcription also proved difficult when quality of Internet connection or participants computer microphones produced poor sound quality. Therefore, there is a small possibility that important data may have been missed. Literature available for online focus group methods is limited, and often refers to typed responses as part of an asynchronous discussion forum [37, 38]. Further research into improving data collection via online synchronous focus groups should be considered.

Clarification of the results from this study could be obtained by a short survey of a larger group to ensure data reliability and empirical generalisability. Focus should also be given to user’s knowledge of databases, as opposed to the software used, and their understanding of database limitations, including the use of supporting documentation. Additionally, a prototype database with data values presented as a range could be trialled with users.

## Conclusion

The ideal format of a FCDB according to user’s would be one in which the database was designed specifically for the intended use, has a user-friendly food classification system, as well as accurate data with a clear explanation of data derivation and the input of users. However, such databases are limited by available resources and data accessibility. Further exploration of data sharing options should be considered. Furthermore, user’s understanding of food composition data and databases limitations is inherent to the correct application of non-specific databases. Therefore, further exploration of user FCDB training should also be considered. This is particularly essential when working with phytochemical data, due to their heightened variability and largely unknown mechanisms of action.

## Supporting Information

S1 Fig Flow chart of thematic analysis process. (DOCX)

S2 Fig Application of schematic analysis to dominant themes extracted from focus groups and interview. (DOCX)

S1 File List of 24 dominant themes. (DOCX)

S1 Table Outline of questions covered in focus groups. (DOCX)

S2 Table Outline of questions covered in interviews. (DOCX)

## Abbreviations

FCDB: Food composition database
