# Supplementary material for: Food Composition Database Format and Structure: A User Focused Approach
Source: PLoS One. 2015 Nov 10;10(11):e0142137. doi: 10.1371/journal.pone.0142137 (PMC4640668; doi:10.1371/journal.pone.0142137)
Supplement: S2 Fig — (DOCX) [file pone.0142137.s002.docx]

Food Classification

Data Derivation

Framework

Accessibility & Availability

Type Purpose Usability Database choice

Central repository

**Format**

Processing Structure Display Storage

**Definition**

**Major Themes**

**Sub-categories**

Database use
