# Supplementary material for: Food Composition Database Format and Structure: A User Focused Approach
Source: PLoS One. 2015 Nov 10;10(11):e0142137. doi: 10.1371/journal.pone.0142137 (PMC4640668; doi:10.1371/journal.pone.0142137)
Supplement: S1 File — (DOCX) [file pone.0142137.s003.docx]

S1 File: List of 24 dominant themes

1. Database use
2. Awareness of limitations
3. Values are approximations
4. Training
5. Data derivation
6. Applicability
7. Resources
8. Accessibility and availability
9. Constantly changing food supply
10. Human variability
11. Food components
12. Framework
13. Food classification
14. Food based guidelines over nutrient based guidelines
15. Public health issues and advocacy
16. Database development
17. Updating
18. Complete/incomplete
19. Data accuracy and quality
20. Need data/database to support research
21. Need evidence before database can be developed
22. Phytochemical definition
23. Effect of food chemicals
24. Mediator interactions and participant examples
