# Supplementary material for: Food Composition Database Format and Structure: A User Focused Approach
Source: PLoS One. 2015 Nov 10;10(11):e0142137. doi: 10.1371/journal.pone.0142137 (PMC4640668; doi:10.1371/journal.pone.0142137)
Supplement: S1 Table — (DOCX) [file pone.0142137.s004.docx]

| Table S1: Outline of questions covered in focus groups |
| --- |
| 1. When you hear the words food composition in general, what thoughts come to mind? |
| 1. What types of food composition training do you recall receiving? |
| 1. What types of food composition resources or activities do you actively seek for your continuing professional development? |
| 1. When you think of food composition in Australia, what thoughts come to mind? |
| 1. What have been your experiences with using Australian food composition databases? |
| 1. When you think of food composition outside of Australia what thoughts come to mind? |
| 1. What have been your experiences with using overseas food composition databases? |
| 1. How frequently would you use a food composition database? |
| 1. For what types of activities would you use a food composition database? |
| 1. What barriers are you faced with when using a food composition database? |
| 1. What comes to mind when you think of food composition database development? |
| 1. If you could, what would you change from Australia’s food composition databases? |
| 1. What comes to mind when you hear the word phytochemical? |
