# Supplementary material for: Food Composition Database Format and Structure: A User Focused Approach
Source: PLoS One. 2015 Nov 10;10(11):e0142137. doi: 10.1371/journal.pone.0142137 (PMC4640668; doi:10.1371/journal.pone.0142137)
Supplement: S2 Table — (DOCX) [file pone.0142137.s005.docx]

| Table S2: Outline of questions covered in key informant interviews |
| --- |
| 1. What have been your experiences with using Australian food composition databases? |
| 1. What barriers are you faced with when using a food composition database? |
| 1. If you could, what would you change from Australia’s food composition databases? |
| 1. Given the mention of phytochemicals in the Australia Dietary Guidelines, do you think it would be useful to have an Australian database for phytochemicals? |
