# Supplementary material for: Food Composition Database Format and Structure: A User Focused Approach
Source: PLoS One. 2015 Nov 10;10(11):e0142137. doi: 10.1371/journal.pone.0142137 (PMC4640668; doi:10.1371/journal.pone.0142137)
Supplement: Supplementary file 5 [file pone.0142137.s001.docx]

Step	Description
Naive read	Inital impression documented individually.
Second read	Transcribed focus groups coded individually.
Code Comparison	Discussion and formation of code names and definitions.
Themes and sub-codes	Combining of codes into themes and sub-categories.
Further coding	Key informant interviews coded as above, with new codes established and documented.
Refining	Codes refined and redefined as neccessary. New themes and sub-categories created and /or merged.
Exemplary quotes extracted	Quotes that exemplefied themes and sub-categories extracted.
Refining	Codes refined and redefined as neccessary. New themes and sub-categories created and/or merged.
Dominant themes	24 dominant themes established.
Schematic analysis	Critically evalute dominant themes using schematic analysis to review themes in line with the research questions and extract major themes individually.
Review sub-themes	Refine and re-evaluate major themes through discussion and review of coded data and quotes under each major theme.
